# Supplementary material for: Fatty acid metabolic reprogramming via mTOR-mediated inductions of PPARγ directs early activation of T cells
Source: Nat Commun. 2016 Nov 30;7:13683. doi: 10.1038/ncomms13683 (PMC5141517; doi:10.1038/ncomms13683)
Supplement: Supplementary Information — Supplementary Figures 1-8 [file ncomms13683-s1.pdf]

a

HMDB ID	Compound Name	Order
HMDB10379	1-Myristoyl-glycero-3-phosphocholine	1
HMDB10385	1-Oleoyl-glycero-3-phosphocholine	2
HMDB10570	1,2-Dipalmitoyl-glycero-3-phosphoglycerol	3
HMDB00207	Oleic acid	4
HMDB03229	Palmitoleic acid	5
HMDB00252	Sphingosine	6
HMDB00067	Cholesterol	7
HMDB00220	Palmitic acid	8
HMDB10382	1-Palmitoyl-glycero-3-phosphocholine	9
HMDB11503	1-Palmitoyl-glycero-3-phosphoethanolamine	10
HMDB002231	cis-11-Eicosenoic acid	11
HMDB00827	Stearic acid	12
HMDB03312	Daidzein	13
HMDB10384	1-Stearoyl-glycero-3-phosphocholine	14
No ID	1-Hexadecyl-2-acetyl-glycero-3-phosphocholine	15
HMDB10169	Sphingomyelin(d18:1/16:0)	16
HMDB00673	Linoleic acid	17
HMDB01043	Arachidonic acid	18
HMDB05060	cis-11,14-Eicosadienoic acid	19
No ID	FA(22:4)	20
No ID	FA(22:5)	21
HMDB11188	Trilaurin	22
HMDB02183	cis-4,7,10,13,16,19-Docosahexaenoic acid	23
HMDB00653	Cholesterol sulfate	24
HMDB01348	Sphingomyelin(d18:1/18:0)	25
No ID	AC(20:1)	26
HMDB08923	1,2-Dipalmitoyl-glycero-3-phosphoethanolamine	27
HMDB08036	1,2-Distearoyl-glycero-3-phosphocholine	28
HMDB00222	Palmitoylcarnitine	29

b

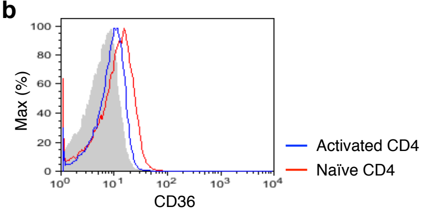

Supplementary Figure 1 (Supplemental data for Figure 1)

(**a**) The list of intracellular lipid metabolites in CD4 T cells described in **Figure 1a.** It is listed in order of higher levels of metabolites in stimulated cells for 48 hours. (**b**) Expression profiles of CD36 on naïve and activated CD4 T cells. Three independent experiments were performed and showed similar results.

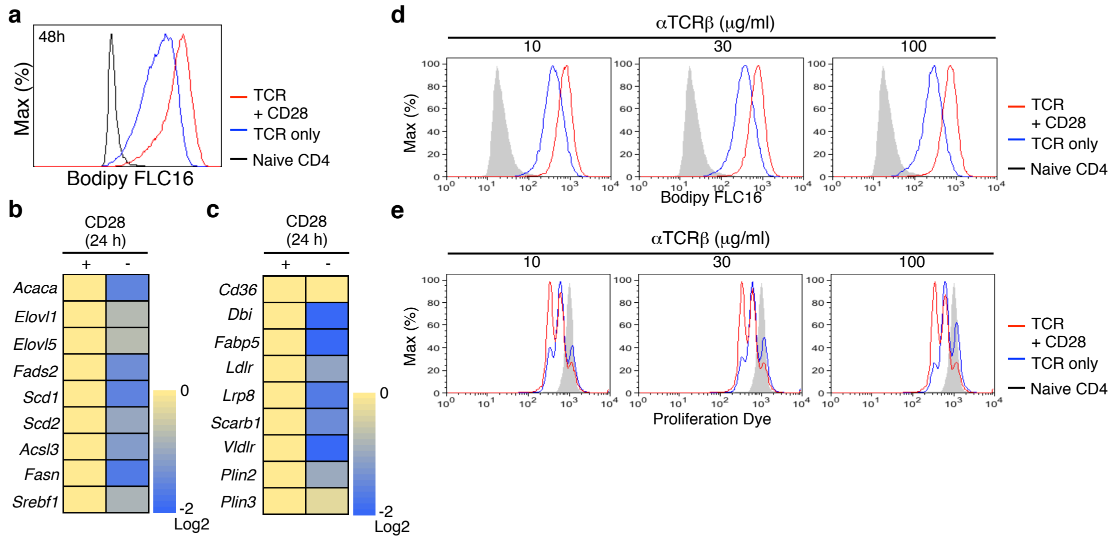

Supplementary Figure 2 (Supplemental data for Figure 2)

(**a**) Representative plots of Bodipy FLC16 in activated CD4 T cells 48 hours after TCR stimulation with or without CD28 co-stimulation are shown. (**b**) qRT-PCR analyses of the relative expression of the genes encoding the enzymes in fatty acid biosynthesis program in activated CD4 T cells 24 hours after TCR stimulation with or without CD28 co-stimulation. The heat map represents the log2 value of the relative mRNA expression level (see color scale). The log2 value of each gene in control cells was set to 0. (**c**) qRT-PCR analyses of the relative expression of the genes encoding the enzymes and transporter in fatty acid uptake program in activated CD4 T cells as in (**b**). (**d**) Representative plots of Bodipy FLC16 in activated CD4 T cells 48 hours after different doses of TCR stimulation with or without CD28

co-stimulation are shown. (**e**) Representative profiles of e670 n activated CD4 T cells 48 hours after different doses of TCR stimulation with or without CD28 co-stimulation are shown. Three technical replicates were performed for qRT-PCR (**b and c**). Three independent experiments were performed with similar results (**a-c**). Two independent experiments were performed with similar results (**d and e**).

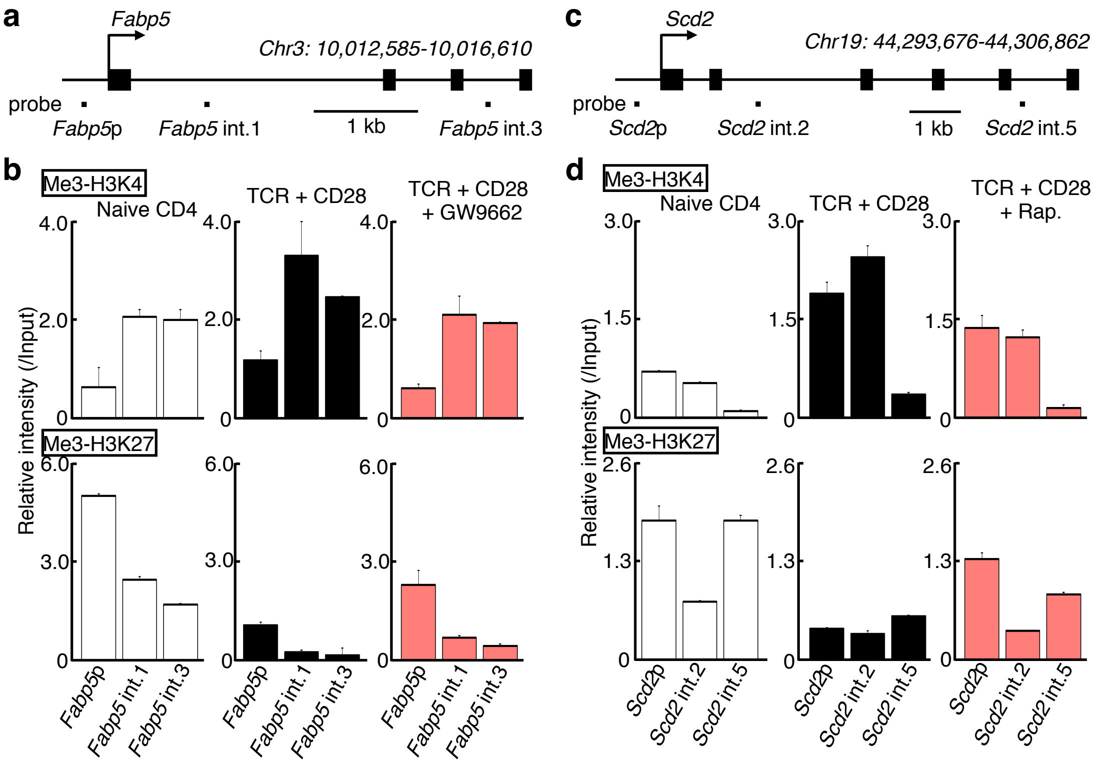

Supplementary Figure 3 (Supplemental data for Figure 3)

(**a**) A schematic representation of the mouse *Fabp5* gene locus. (**b**) ChIP assays were performed with anti-trimethyl histone H3-K4 and anti-trimethyl histone H3-K27 at the *Fabp5* gene locus from naïve CD4 T cells and activated CD4 T cells treated with DMSO (Control) and GW9662 (10 μM). (**c**) A schematic representation of the mouse *scd2* gene locus. (**d**) ChIP assays were performed with anti-trimethyl histone H3-K4 and anti-trimethyl histone H3-K27 at the *Scd2* gene locus from naïve CD4 T cells and activated CD4 T cells treated with DMSO (Control) and

rapamycin (Rap.; 5 nM) as in (b). Three technical replicates were performed for ChIP qRT-PCR (b and d). Three independent experiments were performed with similar results.

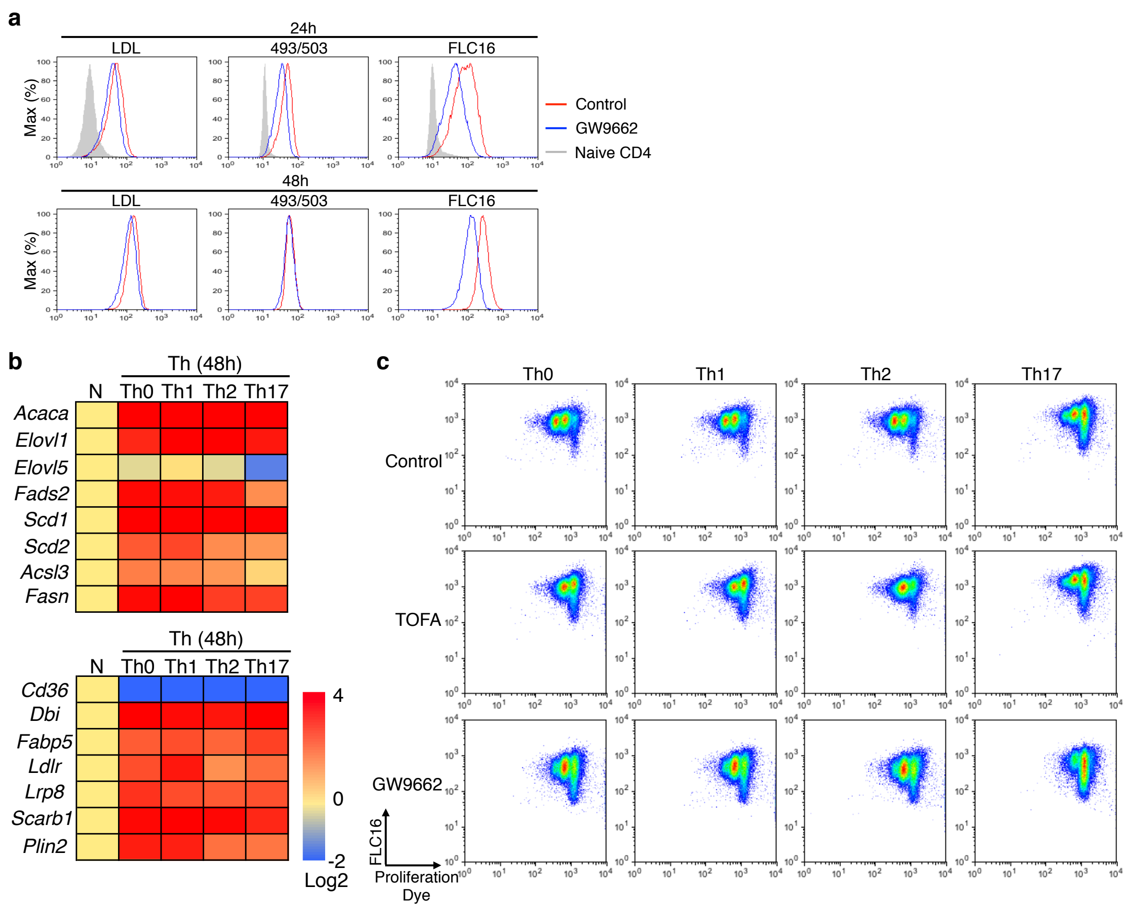

Supplementary Figure 4 (Supplemental data for Figure 4)

(**a**) Representative plots of Bodipy-labeled LDL, Bodipy-labeled 493/503, or Bodipy-labeled FLC16 in activated CD4 T cells 24 or 48 hours after TCR stimulation with or without GW9662 are shown. (**b**) qRT-PCR analyses of the relative expression of the genes encoding the enzymes in fatty acid biosynthesis and fatty acid uptake programs in differentiating Th0, Th1, Th2, and Th17 cells. (**c**) Representative profiles of e670 and Bodipy FLC16 in differentiating Th0, Th1, Th2, and Th17 cells in the presence or

absence of TOFA or GW9662. Three technical replicates were performed for qRT-PCR (**b**). The mean values with standard deviations (SD) are shown. ***P*<0.01 Two independent experiments were performed with similar results (**a**-**c**).

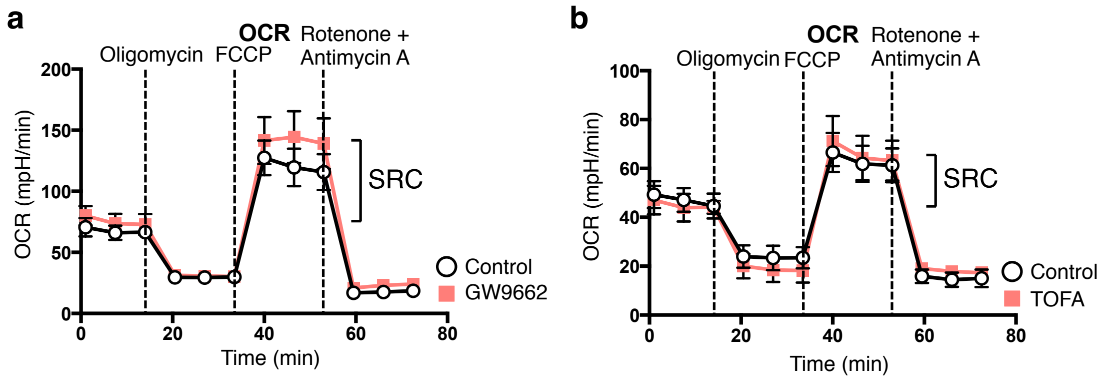

Supplementary Figure 5 (Supplemental data for Figure 5)

(**a**, **b**) OCR of activated memory Th2 cells 48 hours after TCR stimulation with or without GW9662 (**a**) or TOFA (**b**) under basal conditions and in response to sequential treatment with Oligomycin, FCCP and Rotenone-Antimycin A. Six technical replicates were performed for Seahorse assay (**a and b**). The mean values with standard deviations (SD) are shown. Three independent experiments were performed with similar results (**a and b**).

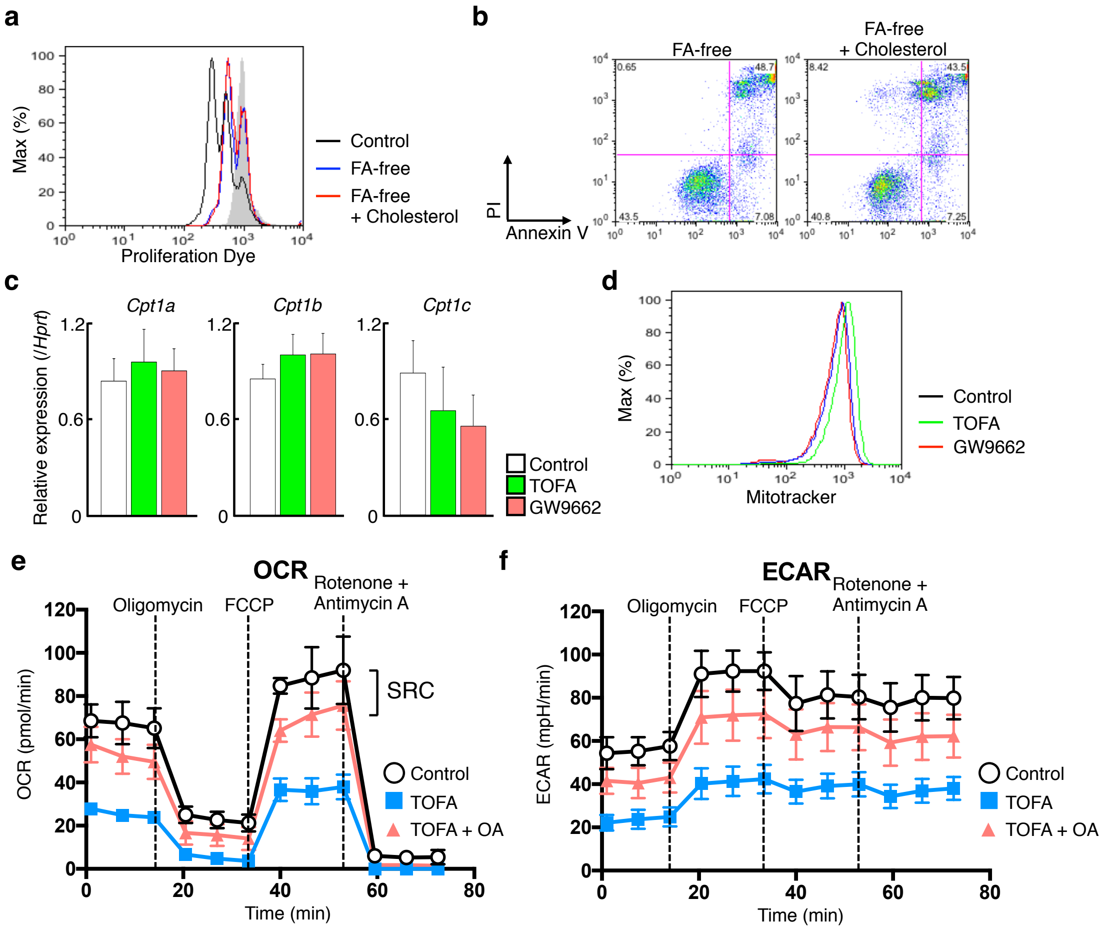

Supplementary Figure 6 (Supplemental data for Figure 6)

(**a**) Representative plot of e670 proliferation dye in activated CD4 T cells with or without cholesterol treatment (5 μg/ml) in normal or fatty acid-free conditions 48 hours after TCR stimulation was shown. (b) Susceptibility to apoptosis of stimulated CD4 T cells was investigated by Annexin V and propidium iodide (PI) staining with similar conditions in the presence or absence of cholesterol (5 μg/ml) in fatty acid-free conditions. (**c**) qRT-PCR analyses of the relative expression of *cpt1a*, *cpt1b*, or *cpt1c* in activated CD4 T cells in the presence or absence of TOFA or GW9662.

(d) Mitotracker staining of activated CD4 T cells in the presence or absence of TOFA or GW9662 was performed by flow cytometry. (e) OCR of activated CD4 T cells 48 hours after TCR stimulation with or without TOFA treatment (10 μM) in the presence or absence of oleic acid (100 μM) under fatty acid-free conditions under basal conditions (Time point 0) and in response to sequential treatment with Oligomycin, FCCP and Rotenone-Antimycin A. (f) ECAR activated CD4 T cells 48 hours after TCR stimulation with or without TOFA treatment (10 μM) in the presence or absence of oleic acid (100 μM) under fatty acid-free conditions under basal conditions and in response to sequential treatment with Oligomycin, FCCP and Rotenone-Antimycin A as in (e). Three technical replicates were performed for qRT-PCR (c). Six technical replicates were performed for Seahorse assay (e and f). The mean values with standard deviations (SD) are shown. Two independent experiments were performed with similar results (a-f).

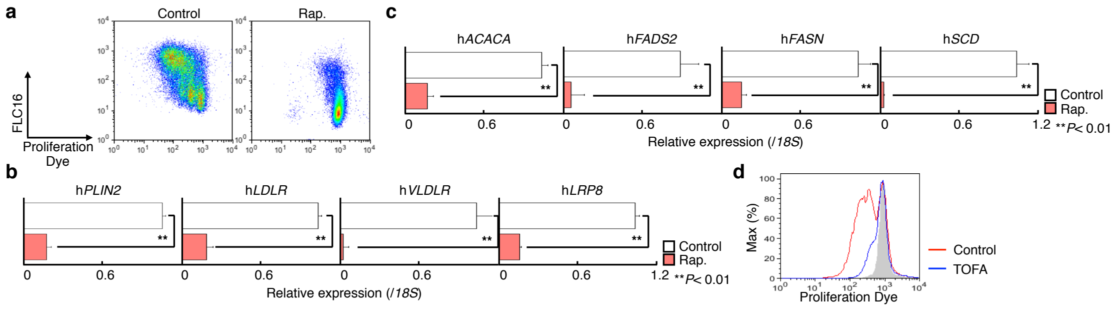

Supplementary Figure 7 (Supplemental data for Figure 7)

(**a**) Naïve CD4 T cells were labeled with e670 proliferation dye and stimulated with an immobilized anti-TCRβ mAb and anti-CD28 mAb in the presence of Bodipy FLC16 with or withouot rapamycin (Rap.: 5 nM). (**b and c**) qRT-PCR analyses of the relative expression of the genes encoding enzymes and transporters in fatty acid uptake programs (**b**) and the fatty acid biosynthesis programs (**c**) and in activated human CD4 T cells in the presence or absence of rapamycin (5 nM). The heat map represents the log2 value of the relative mRNA expression level (see color scale). (**p < 0.01, Mann-Whitney U test) (**d**) Naïve CD4 T cells were labeled with e670 proliferation dye and stimulated with an immobilized anti-TCRβ mAb and anti-CD28 mAb in the presence or absence of TOFA (10 μM). Three independent experiments were performed with similar results (**a-d**).

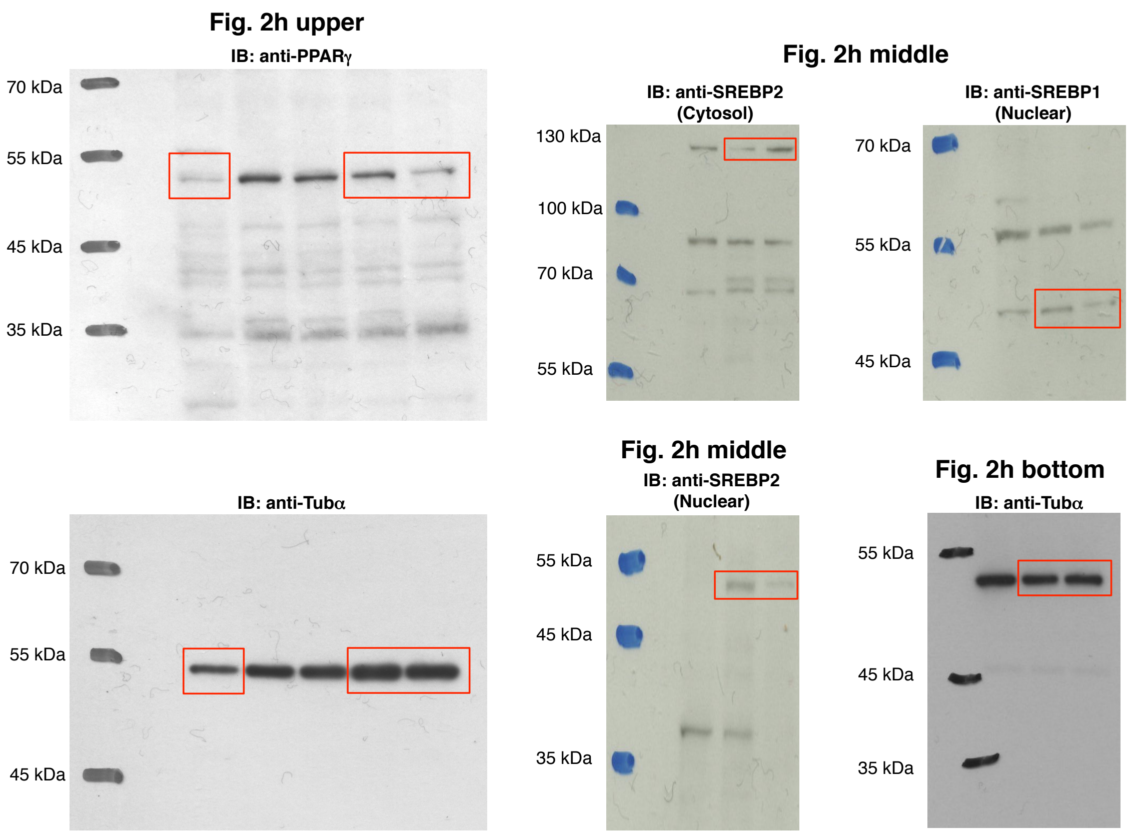

Supplementary Figure 8

Full-size scans of all western blots are shown with labeled and molecular weight ladder.
